# Prevalence of incivility between ophthalmology and emergency medicine residents during interdepartmental consultations

**DOI:** 10.1002/aet2.10653

**Published:** 2021-08-01

**Authors:** Glory E. Mgboji, Fasika A. Woreta, Michael J. Fliotsos, Sidra Zafar, Joseph Ssekasanvu, Divya Srikumaran, Jiawei Zhao, Daniel L. Buccino, Linda Regan

**Affiliations:** ^1^ Johns Hopkins School of Medicine Baltimore Maryland USA; ^2^ Wilmer Eye Institute Johns Hopkins School of Medicine Baltimore Maryland USA; ^3^ Department of Epidemiology Johns Hopkins Bloomberg School of Public Health Baltimore Maryland USA; ^4^ Department of Psychiatry Johns Hopkins University School of Medicine Baltimore Maryland USA; ^5^ Department of Emergency Medicine Johns Hopkins University School of Medicine Baltimore Maryland USA

## Abstract

**Objectives:**

Since incivility is linked to adverse effects in patient care and health care worker well‐being, evaluation of the prevalence of incivility during the formative years of residency training is warranted. The aim of this study was to determine the perceived presence and degree of incivility between emergency medicine (EM) and ophthalmology residents during emergency department (ED) consultations.

**Methods:**

We conducted a single‐site, survey‐based study, targeted to ophthalmology and EM residents. The survey we distributed included questions adapted from validated and widely used surveys measuring incivility in the workplace (Workplace Incivility Scale) and incivility within the ED.

**Results:**

Ophthalmology (13/15, 86.7%) and EM (42/48, 87.5%) residents participated, with an overall response rate of 55 of 63 (87.3%). Most residents (47/55, 85.5%) reported some degree of incivility during consultations, with a greater proportion of females reporting incivility (100%) than males (77.4%, p* *= 0.033). A total of 52.7% of respondents reported occurrence of incivility on a quarterly basis; 21.8% reported monthly, 10.9% weekly, and none daily. Incivilities were reported most commonly during nonurgent consults (85.5%). The two most common incivilities reported by trainees were when the other party paid little attention to their statements or opinions (80% of residents) or doubted their professional judgment (74.5% of residents). More female trainees reported jokes being told at their expense compared to males (15.8% vs. 0%, p* *= 0.049). Residents most often attributed incivility to stress (78.2%), loss of empathy/burnout (63.6%), or attempts to shift responsibility to another party (60.0%). Among EM residents surveyed, incivility was identified as occurring most often during consultations with surgical specialties.

**Conclusions:**

Incivility during interdepartmental consultations between EM and ophthalmology is commonly reported by physicians‐in‐training. It occurs more often during consultations deemed as nonurgent and is more commonly reported by females. Given its associations with adverse outcomes, interventions to decrease incivility early in training may be warranted.

## INTRODUCTION

Incivilities have long been described in the workplace.^1^ The term *workplace incivilities*—defined as “seemingly insignificant behaviors that are rude, disrespectful, discourteous, or insensitive, where the intent to harm is ambiguous or unclear”^2^—is used to describe behaviors such as using blaming or belittling comments, interrupting or ignoring a colleague, or making dismissive remarks or gestures such as eye‐rolling.^3^, ^4^ Workplace incivilities have been shown to negatively impact work performance, impair task‐related memory, and decrease engagement and commitment to one's respective organization.^1^, ^5^ In the realm of health care, incivility has been identified as a threat to patient safety through a degradation of trust among colleagues, weakening the communication and cooperation necessary to provide essential patient care.^6^ Some also believe that workplace incivilities can be linked to medical errors and patient deaths.^7^

Studies have explored incivility in the general workplace,^1^ in the field of nursing,^8^, ^9^ and in the emergency department (ED);^5^ however, little is known about the prevalence of incivilities between physicians and, in particular, among physicians‐in‐training.^10^ As a unique population in the health care setting, physicians‐in‐training (also known as resident physicians) play a significant role in direct patient care across a variety of practice settings. While nurses and students have been identified as at high risk for disrespectful treatment,^6^ residents are also a vulnerable population given their low position in the medical hierarchy. Although simulated operative encounters have found that learners experiencing incivility have decreased performance in diagnosis, communication, and patient management when compared to a control group,^11^ there is no literature focused on the impact of incivilities on trainee well‐being.

Given the limited incivility data among residents and the unique position of our institution as a large referral center for ocular trauma and ophthalmic disease, we aimed to assess the prevalence of incivility between ophthalmology and emergency medicine (EM) residents, a population with a high volume of interdepartmental interactions between residents training in these two specialties. We also aimed to highlight which specific clinical scenarios and interpersonal interactions most often led to incivility in this cohort.

## METHODS

### Study design

We used a single‐center, cross‐sectional survey design to determine the prevalence of incivility between residents in two specialties at our medical center. This study was reviewed by the institutional review board and was considered exempt from review.

### Study setting and population

The study site is a large, urban, academic medical center that serves as the regional eye referral center, with a wide spectrum of medical, surgical, and hospital‐based training specialties. All residents training in both the EM (PGY‐1 to ‐4) and ophthalmology (PGY‐2 to ‐4) residency programs were eligible to participate. No residents were excluded.

### Study tool development

The survey developed was adapted from validated and widely used surveys described by Cortina et al.^12^, ^13^ and Klingberg et al.,^5^ which focused on incivility in the workplace (Workplace Incivility Scale) and incivility within the ED, respectively. Only minor changes were made to question stems to clarify that EM residents should answer the questions specifically about interactions with ophthalmology residents or residents from other subspecialties. Similarly, minor changes were made to the questions to specify that ophthalmology residents should answer the questions about interactions with EM residents or residents from other specialties. For example, the first question stem of the survey administered to EM residents reads, “During the past year, were you ever in a situation in which you called an ophthalmology consult and the consulting resident …” as opposed to the original question stem of “… were you ever in a situation in which your supervisors or coworkers ….” Since the incivility workplace survey developed by Cortina and colleagues has been widely used and validated in occupational settings, we did not feel that it was necessary to pilot before deployment. Incivility was defined for all participants as “low‐intensity, deviant behavior with ambiguous intent to harm the target, in violation of workplace norms for mutual respect.”^13^ The survey collected basic demographic information, including age, sex, race, and year of training. We asked participants about perceived prevalence of incivility between the two services, specifically noting the types of incivility they experienced, perceived causes of incivility, specific situations during which they experienced incivility, and their assessment of how the incivility between ophthalmology and EM compares to other interspecialty relationships they experience. Given the difference in role between EM and ophthalmology residents during consultation, there were slight differences in wording for the surveys given to each group (Data Supplement S1 and S2, Appendixes S1 and S2, available as supporting information in the online version of this paper, which is available at http://onlinelibrary.wiley.com/doi/10.1002/aet2.10653/full). In addition, EM residents were asked to rank the top three consultant specialty departments that, in their experience, were more commonly linked to incivility during the clinical care of patients.

### Study protocol

The study team distributed an anonymous, online survey to all EM and ophthalmology residents at the Johns Hopkins Hospital using an online survey software (Qualtrics). Emails contained a survey link as well as study information. To preserve anonymity, participants were not asked for their names. Instead, each participant was anonymously assigned a unique identifying number that could be used to assess for any future interventions that came from the research. No identifiable data could be linked back to participating residents. Participation in the survey was voluntary, with the decision to participate serving as their consent. Residents were assured that their decision on whether to participate would have no bearing on their employment or evaluation in the residency program and that their program director would not be informed about their participation. The survey was open online for 1 month, with two reminders sent out every 2 weeks after the initial email.

### Key outcome measures

The primary objectives of this study were (1) to determine the perceived presence and degree of incivility between ophthalmology and EM residents and (2) to gain a better understanding of specific situations that may present high‐risk situations and types of behaviors that could most easily be addressed. Secondary objectives included: (1) to stratify where ophthalmology falls in comparison to other consultant specialties regarding incivility perceived by EM residents at our institution and (2) to stratify which consultant departments had the highest degree of incivility for EM residents in our institution. Our hope was to use this information to inform an interspecialty curriculum to improve civility for our trainees.

### Data analysis

Descriptive statistics were used to present demographic data, with age presented as mean (±SD) and self‐identified sex, race, and level of training presented as *n* (%) of the entire cohort. The prevalence, frequency, and perceived factors related to incivility were analyzed. Incivility was considered present for any respondent who answered with a frequency other than “never,” with frequency being recorded by the specific time frames selected. Fisher's exact testing was used to examine differences in the proportion of residents reporting incivility experiences when stratified by the following subgroups: EM versus ophthalmology, male versus female, White versus non‐White, and PGY‐4 (final year of training in both specialties) versus all other years. p‐values < 0.05 were considered to be statistically significant. Because the primary purpose was to present and describe the data rather than test a hypothesis, p‐values for the different outcomes were presented without adjusting for multiple comparisons. Statistical tests were completed using Microsoft Excel (Microsoft Corporation) and Stata Statistical Software (Version 14). Multiple logistic regression was used to estimate adjusted association of each exposure variable (i.e., resident specialty, sex, race, and year of training) with the outcome variable of incivility. Outcome of incivility was determined for this model using a binary composite score that counted any survey response indicating any type of incivility, occurrence of incivility, or poor communication between specialty groups as “yes” for incivility experience.

## RESULTS

A total of 55 of 63 residents participated for an overall response rate of 87.3%; specialty specific response rates were 86.7% (*n* = 13/15) for ophthalmology residents and 87.5% (*n* = 42/48) for EM residents (Table 1). The mean (±SD) age of all respondents was 30.8 (±3.4) years. More than half of participants identified as male (60.8%) and Caucasian (55.1%). There was a significant difference in sex and racial distribution between EM and ophthalmology residents, with a greater proportion of males in the EM group (p = 0.015) and a greater proportion of Caucasian residents in the EM group (p = 0.007). Residents from all 4 years of EM training (and all 3 years of ophthalmology training) were evenly represented in the study population.

**TABLE 1:** Study population characteristics, including number of participants of certain gender, race, and level of training

Characteristic	Total	EM	Ophthalmology
Age (years)	30.8 (±3.4)	31.1 (±3.7)	29.8 (±1.7)
Sex			
Male	31 (56.4)	28 (66.7)	3 (23.1)
Female	20 (36.4)	12 (28.6)	8 (61.5)
Declined	4 (7.3)	2 (4.8)	2 (15.4)
Race			
Caucasian	27 (49.1)	25 (59.5)	2 (15.4)
African‐American	2 (3.6)	2 (4.8)	0 (0.0)
Asian	15 (27.3)	6 (14.3)	9 (69.2)
Hispanic	2 (3.6)	2 (4.8)	0 (0.0)
Other	3 (5.5)	3 (7.1)	0 (0.0)
Declined	6 (10.9)	4 (9.5)	2 (15.4)
Level of training			
PGY‐1	12 (21.8)	12 (28.6)	0 (0.0)
PGY‐2	14 (25.5)	10 (23.8)	4 (30.8)
PGY‐3	13 (23.6)	9 (21.4)	4 (30.8)
PGY‐4	15 (27.2)	11 (26.2)	4 (30.8)
Declined	1 (1.8)	0 (0.0)	1 (7.7)

Note

Data are reported as mean (±SD) or *n* (%) of the entire cohort.

Abbreviation: EM = Emergency medicine, PGY = Post‐graduate year.

**FIGURE 1:**
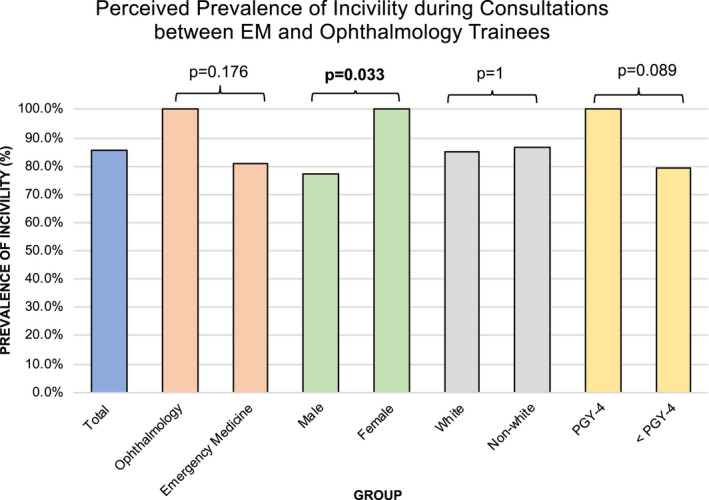
Perceived prevalence of incivility during consultations among trainees. “How often do you feel incivility occurs between EM and ophthalmology residents?” The figure contains the percentage of respondents who noted any amount of incivility among all respondents (*blue*) as well as percentages by specialty (*orange*), sex (*green*), race (*gray*), and year of training (*yellow*). A greater proportion of ophthalmology residents reported incivility than EM (p = 0.176), a greater proportion of females reported incivility than males (p = 0.033); incivility was reported by roughly equal proportions of white versus non‐White residents (p = 1), and a greater proportion of senior residents reported incivility than junior residents (p = 0.089)

Most residents (47/55, 85.5%) reported some degree of incivility during interdepartmental consultations between ophthalmology and EM residents, with the majority reporting a quarterly occurrence. Only 11% reported weekly occurrences and none from either specialty reported daily occurrences. A greater proportion of female trainees reported incivility compared to males (100% vs. 77.4%, p = 0.033, Figure 1). While ophthalmology trainees reported incivility more than EM trainees, and more senior residents (PGY‐4) reported incivility than residents in their earlier years of training (Figure 1), these differences were not statistically significant (p = 0.176 and p = 0.089, respectively). When participants were asked why incivility occurs in this setting, “stress” (78.2%) was the most common answer among both groups. Loss of empathy/burnout (63.6%) and attempts to shift responsibility to another party (60.0%) were also reported as main causes of incivility during consultations between ophthalmology and EM residents (Table 2). Incivilities were overwhelmingly reported as occurring most frequently (85.5%) during nonurgent consults (Table 2).

**TABLE 2:** Survey responses describing prevalence, frequency, and perceived factors related to incivility

	Overall	Ophthalmology	EM
How often do you feel incivility occurs between EM and ophthalmology residents?			
Never	8 (14.5)	0 (0)	8 (19.1)
Rarely (once quarterly)	29 (52.7)	9 (69.2)	20 (47.6)
Occasionally (once a month)	12 (21.8)	2 (15.4)	10 (23.8)
Frequently (once a week)	6 (10.9)	2 (15.4)	4 (9.5)
Very frequently (daily)	0 (0.0)	0 (0.0)	0 (0.0)
Chose not to answer	0 (0.0)	0 (0.0)	0 (0.0)
Compared to other specialties that you interact with, how would you rate the quality of communication between EM and ophthalmology residents?			
Much worse	4 (7.3)	0 (0.0)	4 (9.5)
Worse	6 (10.9)	1 (7.7)	5 (11.9)
Equal	15 (27.3)	5 (38.5)	10 (23.8)
Better	20 (36.3)	5 (38.5)	15 (35.7)
Much better	9 (16.4)	2 (15.4)	7 (16.7)
Chose not to answer	1 (1.8)	0 (0.0)	1 (2.4)
If you experienced incivility in the ED, why do you think incivility occurs? (Select all that apply)			
Stress	43 (78.2)	11 (84.6)	32 (76.2)
Attempts to shift responsibility to another party	33 (60.0)	7 (53.8)	26 (61.9)
Power demonstration	18 (32.7)	3 (23.1)	15 (35.7)
Loss of empathy/burnout	35 (63.6)	8 (61.5)	27 (64.3)
Other, please specify	10 (18.2)	2 (15.4)	8 (19.0)
Chose not to answer	2 (3.6)	0 (0.0)	2 (4.8)
In which situation does incivility most frequently appear? (Select all that apply)			
Acute situations (open globe, retrobulbar hemorrhage)	4 (7.3)	1 (7.7)	3 (7.1)
Obtaining routine (not time‐sensitive) consults	47 (85.5)	12 (92.3)	35 (83.3)
Arranging for a procedure or surgery	2 (3.6)	0 (0.0)	2 (4.8)
Arranging for inpatient admission	8 (14.5)	0 (0.0)	8 (19.0)
Other, please specify	8 (14.5)	1 (7.7)	7 (16.7)
Chose not to answer	3 (5.5)	0 (0.0)	3 (7.1)

Note

Data are reported as *n* (%). Column percentages add to 100% for single‐answer questions

Among all residents, the most common types of incivilities experienced were having statements or opinions overlooked by the other party (reported by 80.0%) or having their judgment doubted on a matter over which they had responsibility (reported by 74.5%, Table 3). A greater proportion of female residents reported jokes being told at their expense than male residents; otherwise, the type of incivility reported did not vary by race, year of training, or specialty (15.5% vs. 0%, p = 0.049, Table 4). Multiple logistic regression was used to estimate adjusted association of each exposure variable (sex, specialty, age, and race) with the outcome variable (whether or not respondents experienced incivility). For the binary composite outcome variable of the presence or absence of incivility, all respondents who stated “no” were male and in EM (providing limited variability with which to analyze the impact of sex and specialty). Due to this, the subsequent multiple regression only included the exposure variables of age and race (see Table S1). Odds of incivility experience did not significantly vary with age (odds ratio [OR] = 0.95, 95% confidence interval [CI] = 0.72 to 1.25) or race (OR = 0.37, 95% CI = 0.03–4.40).

**TABLE 3:** Frequency of type of incivility reported

	Never	Once or twice	Sometimes	Often	Many times	Total
Paid little attention to your statements or showed little interest in your opinions	11 (20)	22 (40)	13 (23.6)	3 (5.5)	6 (10.9)	55
Doubted your judgment on a matter over which you had responsibility	14 (25.5)	22 (40)	13 (23.6)	4 (7.3)	2 (3.6)	55
Made insulting or disrespectful remarks about you	38 (69.1)	11 (20)	3 (5.5)	1 (1.8)	2 (3.6)	55
Addressed you in unprofessional terms, either publicly or privately?	38 (69.1)	12 (21.8)	2 (3.6)	1 (1.8)	2 (3.6)	55
Interrupted or “spoke over” you	36 (66.7)	15 (27.8)	1 (1.9)	2 (3.7)	1 (1.9)	54
Yelled, shouted, or swore at you	50 (94.3)	2 (3.8)	0 (0)	1 (1.9)	0 (0)	53
Targeted you with anger outbursts or “temper tantrums”	44 (83.0)	8 (15.1)	0 (0)	1 (1.9)	0 (0)	53
Made jokes at your expense	50 (92.6)	3 (5.6)	1 (1.9)	0 (0)	0 (0)	54

Note

Data are reported as *n* (%). Rows with less than 55 total responses reflect respondents who declined to answer the question.

**TABLE 4:** Proportions of participants by gender, race, and year of training who reported types of incivility

	Sex			Race			Year of training			Specialty		
	Male	Female	p‐value	White	Non‐White	p‐value	PGY‐4	Non‐PGY‐4	p‐value	EM	Ophtho	p‐value
Paid little attention to your statements or showed little interest in your opinions	24/31	17/20	0.721	21/27	19/22	0.488	14/15	29/39	0.153	34/42	10/13	0.709
Doubted your judgment on a matter over which you had responsibility	23/31	16/20	0.743	20/27	17/22	>0.999	13/15	27/39	0.302	34/42	7/13	0.710
Made insulting or disrespectful remarks about you	11/31	12/20	0.149	7/27	9/22	0.361	7/15	9/39	0.107	13/42	4/13	1
Addressed you in unprofessional terms, either publicly or privately?	9/31	6/20	>0.999	6/27	8/22	0.348	7/15	8/39	0.088	12/42	4/13	1
Interrupted or “spoke over” you	15/31	15/20	0.082	14/27	13/21	0.565	10/14	19/39	0.213	23/41	7/13	1
Yelled, shouted, or swore at you	0/31	2/18	0.130	0/26	1/21	0.447	1/14	2/38	>0.999	1/40	2/13	0.145
Targeted you with anger outbursts or “temper tantrums”	5/31	5/19	0.474	6/27	3/21	0.712	3/14	6/39	0.684	7/41	3/13	0.689
Made jokes at your expense	0/31	3/19	0.049*	1/27	1/22	>0.999	2/14	2/39	0.282	2/41	2/13	0.242

Note

p‐values calculated by Fisher’s exact tests are also displayed.

*Significant p‐values (p < 0.05).

Abbreviation: Ophtho, ophthalmology.

Finally, among EM residents surveyed, ophthalmology placed fourth among subspecialty consultations most commonly linked to incivility. For reference, the top five subspecialties perceived to be commonly linked to incivility by EM residents included neurosurgery (30.1%), urology (16.8%), general surgery (8.9%), ophthalmology (8.0%), and otolaryngology (8.0%; see Table S2).

## DISCUSSION

Incivility in the health care setting is associated with adverse patient care outcomes and negative impacts on physician mental health, including burnout.^6^, ^14^, ^15^ Psychological distress has negative impacts on memory, attention, and overall function,^16^ which impedes effective learning. Despite these important impacts, there is a paucity of literature exploring incivility solely among physicians and physicians‐in‐training.

In 2020, a systematic review examining predictors and triggers of incivility among teams found only eight articles focused solely on physician‐to‐physician incivility, with the remainder of the 53 included studies focusing on nurses or including nurses and other staff in the health care team. Of the eight studies focused on physicians, only three included trainees and none were focused on trainee–trainee interactions.^10^ Our study sought to examine incivility between resident physicians in EM and ophthalmology. While EM physicians are represented to a small extent in the literature, to our knowledge, there are no studies looking at incivility among ophthalmology physicians or trainees in particular. This study aimed to determine the prevalence of incivility between residents in ophthalmology and EM during ED consultations at a regional eye referral center.

The high prevalence of any degree of incivility among trainees in this study builds on our nascent understanding of and draws attention to how incivility is experienced by trainees in medicine. Our trainees experienced incivility with other physicians at frequencies similar to or slightly less than the few studies that have explored this topic.^5^, ^17^ Unfortunately, the literature on incivility may also be blurred or unclear due to the lack of differentiation between specific actions attributed to incivility and discrimination, harassment or “disruptive physician” behaviors,^18^, ^19^ which can range from incivility to frank physical assault. One study found that more than half of surveyed ophthalmology trainees in Australia and New Zealand reported bullying, harassment, or discrimination during their training,^20^ while two studies examining EM physicians noted that junior physicians (both junior faculty and residents) reported that they experienced more incivility than their more senior counterparts.^5^, ^21^ Our cohort, when stratified by residents in their final year of training versus those in the first 3 years, did not appear to report any difference in their experience with incivility. Unfortunately, incivility does not appear to go away after training or with more time as a physician. A study by Mullan et al. in 2013,^22^ which compared disruptive behavior as reported by 2 years of interns across specialties to faculty at one U.S. medical center, found that interns experienced much higher levels of certain disruptive behaviors that could be classified under the domain of incivility than more senior physicians (e.g., condescending behavior 74.6% vs. 35%; berating 20.3% vs. 5%; p < 0.001). However, approximately one‐quarter of both groups reported experiencing similar levels of other types of behaviors (e.g., yelling/raising voice, making inappropriate jokes). It is possible that the type of incivility experienced may change with time, but incivility itself appears to persist.

While a greater proportion of females reported incivility than males in this study, the literature reports mixed findings on the relationship between sex and incivility in health care, with some studies describing an association^21^, ^23^, ^24^, ^25^, ^26^ and others describing no relationship.^20^, ^22^, ^27^, ^28^ The psychology and business management literature has done some work to better understand how incivility affects females in the workplace, with authors proposing that females may have a lower threshold for what they perceive as incivility based on their acculturated norms.^29^ Additionally, it has been theorized that that when faced with unchecked incivility in the workplace, they are more likely to withdraw from work and work‐related tasks, a behavior not seen as often in males.^30^ The disproportionate exposure of females to incivility,^10^ in frequency (as was the case in this study) or along with other concomitant discrimination, may play a role in the sex‐based differences seen in response to incivility. The discrepancies identified in our study warrant further scrutiny to help ensure a positive environment for all trainees, regardless of sex.

Recent literature on rude and disrespectful behaviors in the health care setting report that situational factors related to incivilities have been understudied.^10^ The most common situational context in which incivility was experienced by our trainees was during nonurgent consultations, rather than acute situations. This suggests that incivility occurs even in the absence of the obvious stressors associated with intense clinical interactions and perhaps is reflective of the overall “stress” being experienced by the trainees during their time in residency. Our trainees reported stress and loss of empathy/burnout as the two most common reasons for why incivility occurs. Within EM, one study investigated etiologic factors for incivility within a group of EM physicians in Switzerland; the top two reasons cited were to unload stress and as a demonstration of power.^5^ Heavy workload, communication issues, safety concerns, and nonoptimal team dynamic have all been implicated as situational factors that contribute to incivilities in the health care setting.^5^, ^10^, ^15^, ^17^, ^31^, ^32^

Outside of situational factors leading to incivility, certain specialties have been linked to higher degrees of incivility.^10^ From the perspective of our EM residents, the surgical specialties were most commonly linked to incivility. The top five groups reported were neurosurgery, urology, general surgery, ENT, and ophthalmology. While other studies have named surgical specialties among the domains with more incivilities,^18^, ^26^, ^33^ several other specialties including cardiology^17^ and radiology^19^, ^34^ have also been identified. In addition, from the perspective of our ophthalmology residents, 100% reported some degree of incivility in their consultations with EM, which, although not statistically significant, is higher than the 80% reported by EM residents. It is unclear if any of these findings are based on institutional bias or selection bias or if they reflect a specialty trend. Individual institutional study may be needed to identify and examine the situations where interdepartmental incivility is most likely to arise within a specific health care setting.

Our study highlights the prevalence of incivilities among U.S. EM and ophthalmology trainees and provides a foundation for future study and intervention in this population as well as for other EM‐consultant populations. Given the detrimental impact of incivilities in the health care setting,^6^, ^14^ interventions are warranted to combat them. Positive work atmosphere,^28^, ^35^ diversity,^36^ good leadership,^37^ increased reporting,^15^ and zero tolerance policies for exceedingly disrespectful behaviors^5^ have all been either associated with or suggested for a decrease in incivility. Our findings on the prevalence of incivilities in this population, its presence during nonurgent consultations which might otherwise be thought to be less intense or stressful, and postulated etiologies provide valuable insight on which to build targeted interventions at reducing incivility and provide a baseline understanding of this issue for future work.

## LIMITATIONS

This study has several important limitations to consider. As a single‐institution study, and a regional eye referral center, results may reflect institution‐specific trends that might not be broadly generalizable. The collection of retrospective data using a questionnaire methodology leaves the study vulnerable to recall bias. Additionally, the larger percentage of males in the EM program and females in the ophthalmology program, as well as the racial differences between the groups, may have influenced the responses found given the possibility that females or non‐White trainees may have differing experiences with incivility.^10^ The small sample size of our study population limited our ability to perform statistically meaningful multiple logistic regression models to assess the impact that demographics may have played on trainees’ experience with incivility. Despite these limitations, we believe this study provides valuable insight into interdepartmental interactions among medical trainees and identifies opportunities for future research and intervention.

## CONCLUSIONS

Incivility occurred regularly between ophthalmology and emergency medicine residents and was most often reported during nonurgent consultations and more frequently by females. Stress was reported by both groups as the most common reason why trainees believed incivility occurred. Given the negative impact of incivility on mental health and performance, further study of this topic among trainees across institutions is warranted, with the goal of informing interventions at formative stages, such as medical school and residency.

## CONFLICT OF INTEREST

The authors have no potential conflicts to disclose.

## AUTHOR CONTRIBUTIONS

Study concept and design—Linda Regan, Fasika A. Woreta, Daniel L. Buccino. Acquisition of data—Sidra Zafar, Linda Regan. Analysis and interpretation of data—Glory E. Mgboji, Fasika A. Woreta, Michael J. Fliotsos, Joseph Ssekasanvu, Linda Regan. Drafting of the manuscript—Glory E. Mgboji, Michael J. Fliotsos, LR. Critical revision of the manuscript for intellectual content—all authors. Acquisition of funding—Fasika A. Woreta, Linda Regan. Statistical expertise –Joseph Ssekasanvu.

## Supporting information

**Data Supplement S1**. Supplemental material.
