# Supplementary material for: Prevalence of incivility between ophthalmology and emergency medicine residents during interdepartmental consultations
Source: AEM Educ Train. 2021 Aug 1;5(4):e10653. doi: 10.1002/aet2.10653 (PMC8427183; doi:10.1002/aet2.10653)
Supplement: Supplementary file 1 — Data Supplement S1. Supplemental material. [file AET2-5-e10653-s001.pdf]

Data Supplement S1. Supplemental material

APPENDIX S1: Incivility survey distributed to EM residents.

We are conducting a survey for Emergency Department (ED) residents regarding incivility in the ED among resident consultants. Please take a few minutes to complete the survey. All responses will be anonymous and only aggregate data will be shared.

For the purposes of this survey: Incivility is based on the definitions and concepts of Cortina et al and is defined as "low-intensity deviant behaviour with ambiguous intent to harm the target, in violation of workplace norms for mutual respect. Uncivil behaviours are characteristically rude and discourteous, displaying a lack of regard for others"

This definition has been widely used and validated in occupational settings.[1]

We have chosen a unique number for you. Please record it for the duration of this study. Once your data is matched, we will remove your unique ID number.

Your completion of the survey will be considered as consent.

Unique Identifier Number:

For questions 1 and 2, respondents can choose one of the following for each situation: Never, once or twice, sometimes, often, many times, or N/A

1. During the past year, were you ever in a situation in which you called an **ophthalmology consult** and the resident:
    a. Paid little attention to your statements or showed little interest in your opinions
    b. Doubted your judgment on a matter over which you had responsibility
    c. Made insulting or disrespectful remarks about you
    d. Addressed you in unprofessional terms, either publicly or privately
    e. Interrupted or “spoke over” you
    f. Yelled, shouted, or swore at you
    g. Targeted you with anger outbursts or “temper tantrums”
    h. Made jokes at your expense
2. During the past year, were you ever in a situation in which you called a consult **(other than ophthalmology**) and the resident:
    a. Paid little attention to your statements or showed little interest in your opinions
    b. Doubted your judgment on a matter over which you had responsibility
    c. Made insulting or disrespectful remarks about you
    d. Addressed you in unprofessional terms, either publicly or privately
    e. Interrupted or “spoke over” you
    f. Yelled, shouted, or swore at you
    g. Targeted you with anger outbursts or “temper tantrums”
    h. Made jokes at your expense

3. How often do you feel incivility appears between **EM** and **ophthalmology residents**?
Never
Rarely (once quarterly)
Occasional (once a month)
Frequent (once a week)
Very frequently (daily)

4. Compared to other specialties that you interact with, how would you rate the quality of communication between **EM** and **ophthalmology residents**?
Much worse
Worse
Equal
Better
Much better

5. If you have experienced incivility **in the ED**, why do you think incivility occurs? (Select all that apply)
Stress
Using the emergency department as scapegoat
Attempts to shift responsibility to another party
Power demonstration
Loss of empathy/burnout
Other, please specify: ____

6. In which situation does incivility mostly appear with residents **from ophthalmology residents**? (Select all that apply)
Acute situations (open globe, retrobulbar haemorrhage)
Obtaining routine (not time-sensitive) consult
Arranging for procedure/surgery
Arranging for inpatient admission
Other, please specify: _____

7. In which situation does incivility mostly appear with residents from **other departments**? (Select all that apply)
Acute situations
Obtaining routine specialty consult in ED
Arranging for procedure/surgery
Arranging for inpatient admission
Other, please specify: _____

8. Which departments do you think are mostly responsible for incivility? (Please write the top 3)

Ophthalmology
Dermatology
Orthopedic surgery
Urology
ENT
General surgery
Neurosurgery
Vascular surgery
Plastics
Cardiology
Nephrology
GI
Infectious Disease
Neurology
Rheumatology
Anesthesia
OB/GYN

Psychiatry

Most common ________________________
Second most common ________________
Third most common _________________

9. What is your age in years?

10. What is your gender?
Male
Female
Non-gender conforming/gender fluid
Prefer not to answer

11. What ethnicity/ethnicities do you self-identify as (Choose all that apply)?
Caucasian
African American/Black
Asian
Hispanic or Latino
Other (add free text)
Prefer not to answer

12. What is your level of training?
PGY-1
PGY-2
PGY-3
PGY-4

References

1. Cortina LM, Magley VJ, Williams JH, Langhout RD. Incivility in the workplace: incidence and impact. *Journal of occupational health psychology.* 2001;6(1):64-80.

APPENDIX S2: Incivility survey distributed to ophthalmology residents.

We are conducting a survey for Ophthalmology residents regarding incivility in the Emergency Department (ED) among your consultant interactions with the Emergency Medicine (EM) residents. Please take a few minutes to complete the survey. All responses will be anonymous and only aggregate data will be shared.

For the purposes of this survey: Incivility is based on the definitions and concepts of Cortina et al and is defined as "low-intensity deviant behaviour with ambiguous intent to harm the target, in violation of workplace norms for mutual respect. Uncivil behaviours are characteristically rude and discourteous, displaying a lack of regard for others"

This definition has been widely used and validated in occupational settings.[1]

We have chosen a unique number for you. Please record it for the duration of this study. Once your data is matched, we will remove your unique ID number.

Your completion of the survey will be considered as consent.

Unique Identifier Number:

For questions 1 and 2, respondents can choose one of the following for each situation: Never, once or twice, sometimes, often, many times, or N/A

1. During the past year, were you ever in a situation in which you were called for an **ophthalmology consult** by **the EM resident** and the resident:
    a. Paid little attention to your statements or showed little interest in your opinions
    b. Doubted your judgment on a matter over which you had responsibility
    c. Made insulting or disrespectful remarks about you
    d. Addressed you in unprofessional terms, either publicly or privately
    e. Interrupted or "spoke over" you
    f. Yelled, shouted, or swore at you
    g. Targeted you with anger outbursts or "temper tantrums"
    h. Made jokes at your expense
2. During the past year, were you ever in a situation in which you were called for an **ophthalmology consult** by **a non-ED resident** and the resident
    a. Paid little attention to your statements or showed little interest in your opinions
    b. Doubted your judgment on a matter over which you had responsibility
    c. Made insulting or disrespectful remarks about you
    d. Addressed you in unprofessional terms, either publicly or privately
    e. Interrupted or "spoke over" you
    f. Yelled, shouted, or swore at you
    g. Targeted you with anger outbursts or "temper tantrums"
    h. Made jokes at your expense

3. How often do you feel incivility appears between **EM** and **ophthalmology residents**?
Never
Rarely (once quarterly)
Sometimes (once a month)
Frequent (once a week)
Very frequently (daily)

4. Compared to other specialties that you interact with e.g. medicine, neurology, neurosurgery, how would you rate the quality of communication between **EM** and **ophthalmology residents**?
Much worse
Worse
Equal
Better
Much better

5. If you have experienced incivility **in the ED**, why do you think incivility occurs? (Select all that apply)
Stress
Leaving the emergency department as scapegoat
Attempts to shift responsibility to another party
Power demonstration
Loss of empathy/burnout
Not evaluable
Other, please specify: ____

6. In which situation does incivility mostly appear with **EM residents**? (Select all that apply)
Acute situations (open globe, retrobulbar hemorrhage)
Performing routine (not time-sensitive) consult in ED
Arranging for procedure/surgery
Arranging for inpatient admission
Other, please specify: _____

9. What is your age in years?

10. What is your gender?
Male
Female
Non-gender conforming/gender fluid
Prefer not to answer

11. What ethnicity/ethnicities do you self-identify as (Check all that apply)?
Caucasian
African American/Black
Asian
Hispanic or Latino
Other (add free text)
Prefer not to answer

12. What is your level of training?
PGY-1
PGY-2
PGY-3
PGY-4

References
1. Cortina LM, Magley VJ, Williams JH, Langhout RD. Incivility in the workplace: incidence and impact. Journal of occupational health psychology. 2001;6(1):64-80.

2. Klingberg K, Gadelhak K, Jegerlehner SN, Brown AD, Exadaktylos AK, et al. (2018) Bad manners in the Emergency Department: Incivility among doctors. PLOS ONE 13(3): e0194933

Table S1. Multiple logistic regression for odds of incivility experience using composite score of survey responses for incivility outcome.

Variable	Odds Ratio	P value	95% Confidence Interval
Age	0.95	0.727	0.72 – 1.25
Race	0.37	0.431	0.03 – 4.40

Table S2. Specialties linked to incivility, from the perspective of EM residents. The five specialties most frequently chosen are bolded.

**Specialty**	**N (%)**
**Neurosurgery**	34 (30.1)
**Urology**	19 (16.8)
**General Surgery**	10 (8.9)
**Otolaryngology**	9 (8.0)
**Ophthalmology**	9 (8.0)
Neurology	8 (7.1)
Gastroenterology	6 (5.3)
Orthopedic Surgery	5 (4.4)
OB/GYN	5 (4.4)
Plastics	4 (3.5)
Vascular Surgery	2 (1.8)
Rheumatology	1 (0.9)
No department is more uncivil than the other	1 (0.9)
Cardiology	0 (0.0)
Nephrology	0 (0.0)
Anesthesia	0 (0.0)
Infectious Disease	0 (0.0)
Dermatology	0 (0.0)
Psychiatry	0 (0.0)
Total	113 (100)
